# Ipragliflozin attenuates non-alcoholic steatohepatitis development in an animal model

**DOI:** 10.1371/journal.pone.0261310

**Published:** 2022-02-22

**Authors:** Asahiro Morishita, Tomoko Tadokoro, Shintaro Fujihara, Hisakazu Iwama, Kyoko Oura, Koji Fujita, Joji Tani, Kei Takuma, Mai Nakahara, Tingting Shi, Reiji Haba, Keiichi Okano, Akira Nishiyama, Masafumi Ono, Takashi Himoto, Tsutomu Masaki

**Affiliations:** 1 Department of Gastroenterology and Neurology, Kagawa, Japan; 2 Life Science Research Center, Kagawa, Japan; 3 Department of Pathology, Kagawa, Japan; 4 Department of Gastroenterological Surgery, Kagawa, Japan; 5 Department of Pharmacology, Kagawa University Faculty of Medicine, Kagawa, Japan; 6 Department of Medical Technology, Kagawa Prefectual University of Health Sciences, Kagawa, Japan; University of Navarra School of Medicine and Center for Applied Medical Research (CIMA), SPAIN

## Abstract

Non-alcoholic steatohepatitis (NASH) is a common chronic liver disease with no decisive treatment. The sodium glucose cotransporter 2 (SGLT2) inhibitor ipragliflozin was developed as a new oral hypoglycemic drug, which can improve NASH via an insulin-independent glucose-lowering effect by inhibiting glucose reabsorption in the renal proximal tubules. However, ipragliflozin appears to modulate steatosis or inflammation via different pathways. To elucidate the new mechanism of ipragliflozin for the treatment of NASH, we evaluated its effects in a NASH mouse model (STAM mice) with beta cell depletion, and compared the expression of microRNAs (miRNAs) in STAM mice treated with or without ipragliflozin (16.7 μg/day for 5 weeks). Ipragliflozin reduced aspartate transaminase and alanine aminotransferase levels, along with reduced hepatic steatosis, hepatocyte ballooning, lobular inflammation, and liver fibrosis. In addition, ipragliflozin upregulated mitochondrial transport-related and antioxidant defensive system-related genes in the liver. Among 2555 mouse miRNA probes, miR-19b-3p was commonly differentially expressed with ipragliflozin treatment for 5 weeks in both the liver and serum but in different directions, with a decrease in the liver and increase in the serum. Therefore, ipragliflozin can improve NASH development likely through the antioxidative stress pathway and by regulating miR-19b-3p.

## 1. Introduction

Non-alcoholic steatohepatitis (NASH) is the advanced stage of non-alcoholic fatty liver disease (NAFLD) and is one of the most common chronic liver diseases worldwide with a global prevalence of approximately one-fourth of the adult population [1]. NAFLD has been recognized as the hepatic manifestation of the metabolic syndrome, whose major components are comorbidities such as obesity and type 2 diabetes mellitus (T2DM) [2–4]. In fact, liver diseases, including liver cirrhosis and hepatocellular carcinoma (HCC), represent the third leading cause of death among T2DM patients in Japan [5]. NAFLD comprises various pathological conditions ranging from simple steatosis to NASH, cirrhosis, and HCC [6]. Various drugs such as vitamin E [7, 8], peroxisome proliferator-activated receptor gamma agonists [9, 10], ursodeoxycholic acid [11, 12], antioxidants [11], insulin-sensitizing agents, glucose-lowering drugs [13–18], and lipid-lowering drugs [19–21] have been used for the treatment of NASH. Despite numerous clinical trials and tremendous efforts, there is still no definitive therapy for NASH. Lifestyle alterations, such as exercise and diet control, are currently one of the most effective therapies for NASH [22]. Although such changes bring about better outcomes, they are hard to achieve and maintain for the majority of NASH patients, resulting in poor condition requiring pharmacological therapy.

Sodium glucose cotransporter 2 (SGLT2) inhibitors have been developed as a new class of oral hypoglycemic drugs that inhibit glucose reabsorption at the proximal tubules for the treatment of T2DM [23, 24]. The oral selective SGLT2 inhibitor ipragliflozin has been shown to improve hyperglycemia, dyslipidemia, liver steatosis, oxidative stress, inflammation, and liver injury in T2DM animal models [25–27]. The mechanism of ipragliflozin in the treatment of NASH is mainly considered to be an insulin-independent glucose-lowering effect with subsequent caloric loss via the inhibition of glucose reabsorption in the renal proximal tubules [25–27]. Komiya et al. [18] demonstrated that ipragliflozin also promotes epididymal fat accumulation without worsening adipose inflammation and inhibits ectopic fat accumulation in the liver. This suggested that ipragliflozin is involved in other pathways to modulate steatosis or inflammation, including energy homeostasis and balance between adipose and non-adipose tissues.

MicroRNAs (miRNAs) are 18–22-nucleotide-long interfering, endogenous noncoding RNAs, which are abundant in the human genome, including over 1000 reported miRNA sequences [28]. MiRNAs have broad effects in regulating the expression of various genes, in which one miRNA can target and modulate more than 200 genes [29]. Several reports have recently demonstrated that the miRNA expression profile in the liver is associated with the progression of liver diseases, such as liver fibrosis, cirrhosis, and HCC [30–34]. However, what the effect of ipragliflozin is associated with miRNA expression in the NASH liver remains unexplored.

Therefore, the aim of the present study was to elucidate the effect and mechanism of ipragliflozin for the treatment of NASH using a miRNA array in a mouse model to identify differentially expressed miRNAs in NASH with and without ipragliflozin treatment.

## 2. Results

### 2.1. Biochemical and histological analyses

**Fig 1:**
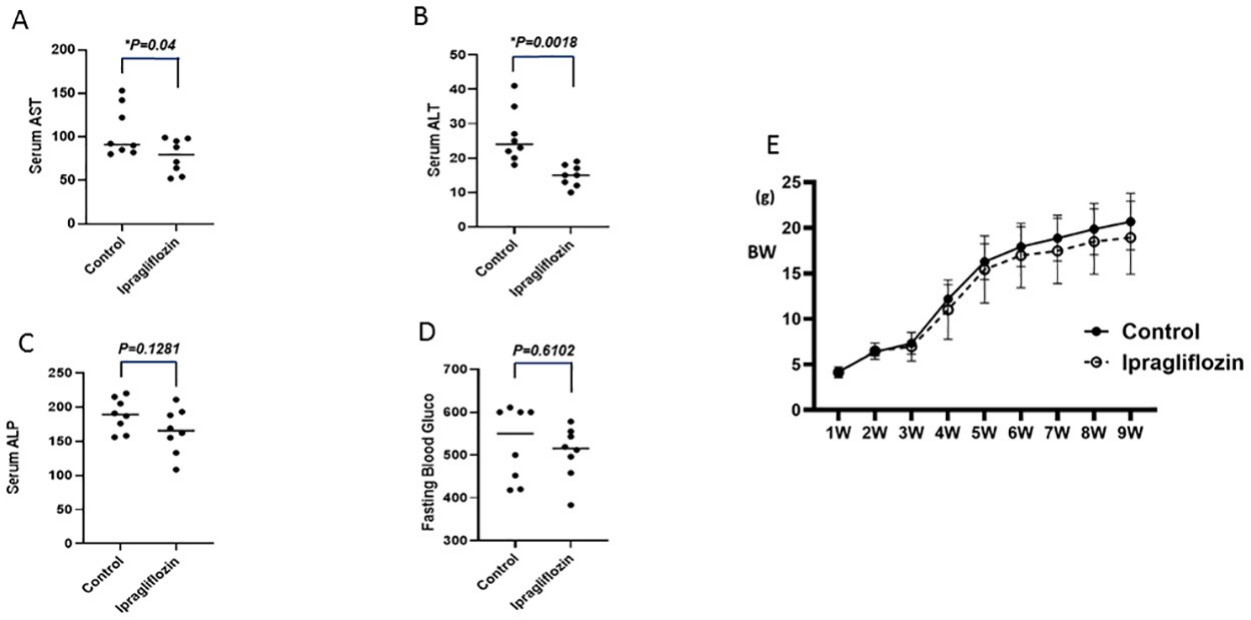
Effects of ipragliflozin on (A) ALT, (B) AST, (C) ALP, (D) fasting blood glucose, and (E) body weight (BW) in the livers of STAM mice. Data are shown as the mean ± SD [*P < 0.025 vs. control group (n = 8) or ipragliflozin group (n = 8)].

Serum AST and ALT levels were significantly reduced in the ipragliflozin group as compared to those of the control group (Fig 1A and 1B). In addition, serum ALP levels were slightly lower in the ipragliflozin group, although the difference was not statistically significant (Fig 1C). Fasting blood glucose levels were very high in both groups bcause pancreatic beta cells were destroyed by the streptozotocin treatment in establishment of this model (Fig 1D). Body weights between control and ipragliflozin groups were not changed (Fig 1E).

**Fig 2:**
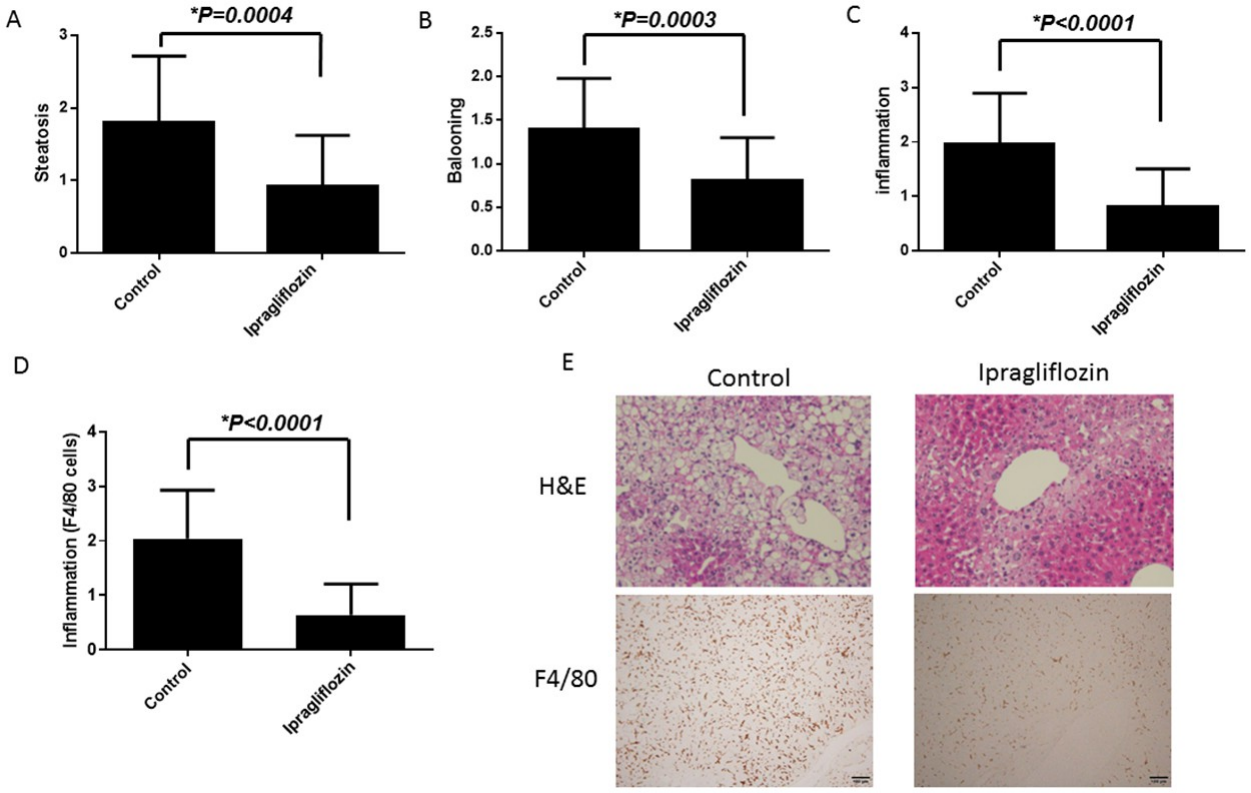
Livers of the STAM mice exhibited severe steatosis, many ballooning hepatocytes, and severe inflammation. Ipragliflozin attenuated the development of hepatic steatosis (A), hepatocyte ballooning (B), lobular inflammation (C) and lobular infiltration of F4/80 positive cells (D). Data are shown as the mean ± SD (*P < 0.05). (E) Paraffin-embedded sections of the liver stained with hematoxylin and eosin and immunohistochemistry by F4/80 antibody (original magnification, 100×). (F) Paraffin-embedded sections stained with Masson’s trichrome (original magnification, 200×). (F, G, H) Ipragliflozin significantly attenuated liver fibrosis. Image analysis of the Masson’s trichrome-stained liver sections was performed using an ImageJ software. Data are shown as the mean ± SD [*P < 0.05 vs. control group (n = 8) or ipragliflozin group (n = 8)].

As shown in Fig 2, the control group developed hepatocyte steatosis, ballooning, and scattered inflammatory cell infiltration including F4/80 positive cells at 10 weeks, which were all clearly reduced in the ipragliflozin group (Fig 2A–2E). The NAFLD activity score was also significantly lower in the ipragliflozin group (Fig 2A–2C).

In addition, liver fibrosis was also significantly diminished in the liver tissues of the ipragliflozin group as compared with those of the control group (Fig 2F–2H). We also confirmed the effect of ipragliflozin for the reduction of liver fibrosis using NAFLD fibrosis score (S1 Fig).

### *2*.2. Ipragliflozin upregulated antioxidative genes and inflammatory markers

**Fig 3:**
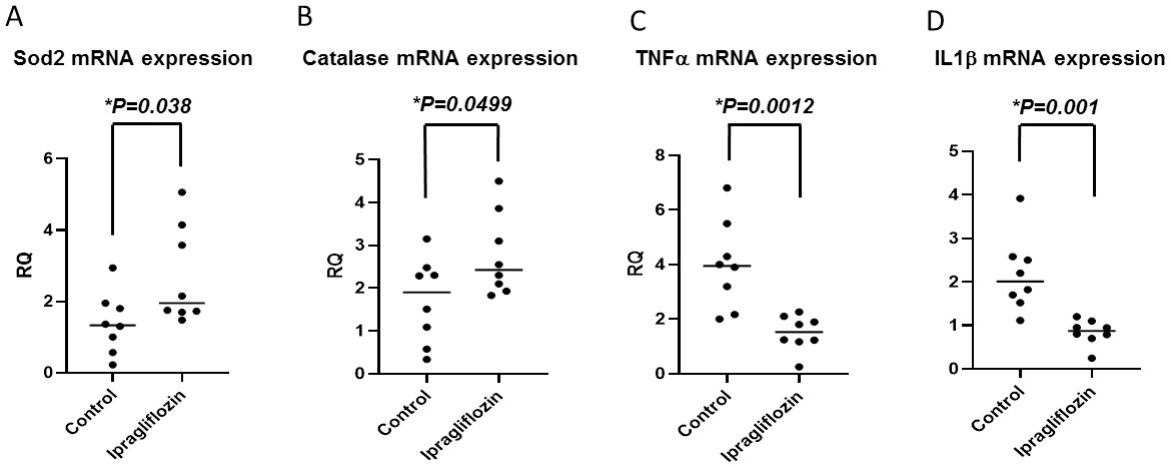
Ipragliflozin enhanced the antioxidative effect in the liver tissue of STAM mice. Levels of mitochondrial transport-related and antioxidant defensive system-related genes (A) *Sod2*, (B) *Cat*, (C) *TNFα*, and (D) *IL1β* in the ipragliflozin (n = 8) and control groups (n = 8).

We further examined the effect of ipragliflozin on oxidative stress in the liver tissues. Ipragliflozin upregulated the expression of mitochondrial transport-related and antioxidant defensive system-related genes in the liver. Specifically, the mRNA levels of *Sod2* and *Cat* were significantly increased in the ipragliflozin group relative to those of the control group (Fig 3A and 3B). In addition, the mRNA levels of *Tnf* and *Il1b* were significantly decreased in the ipragliflozin group relative to those of the control group (Fig 3C and 3D). These results suggest that ipragliflozin improves NASH development through the antioxidative stress and anti-inflammation pathway.

### *2*.3. miRNA expression in the liver tissue and serum samples of STAM mice

**Fig 4:**
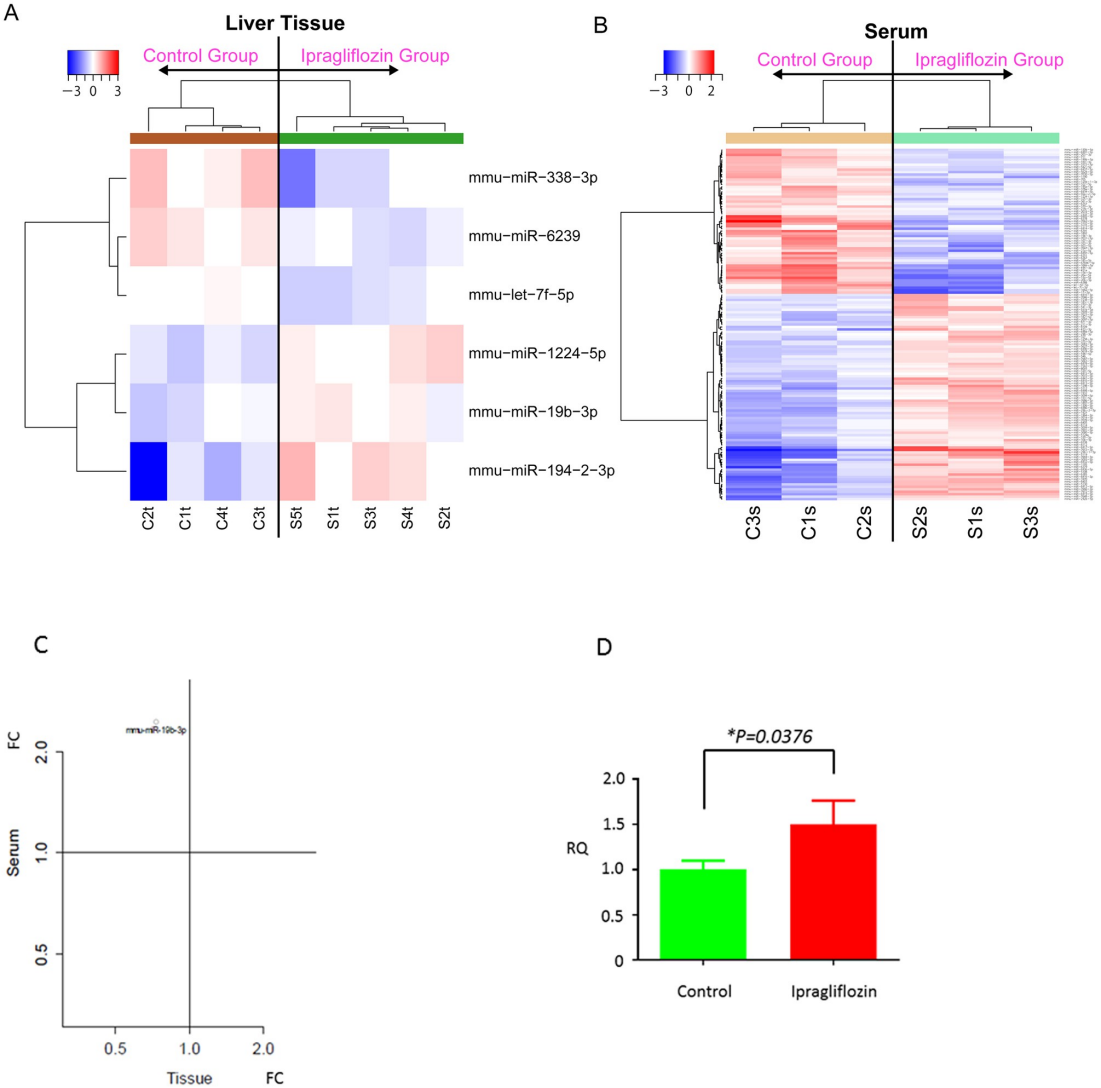
Hierarchical clustering of the liver tissue (A) and serum (B) from STAM mice between the control and ipragliflozin groups. Liver tissues and sera clustered according to the expression profiles of six differentially expressed miRNAs between groups. The analyzed samples are reported in columns and the miRNAs are presented in rows. The miRNA clustering tree is shown on the left, and the sample clustering tree appears at the top. The color scale shown at the top indicates the relative expression level of miRNAs, with red representing a high expression level and blue representing a low expression level. (C) Among 2555 miRNAs analyzed, miR-19b-3p was significantly upregulated in the serum and downregulated in the liver tissue of STAM mice after ipragliflozin administration. (D) RT-qPCR results showing that miR-19b-3p expression was significantly upregulated in the serum of STAM mice treated with ipragliflozin. Data are shown as the mean ± SD [*P < 0.05 vs. control group (n = 8) or ipragliflozin group (n = 8)].

Finally, to uncover the miRNA profiles during the development of NASH, we analyzed the expression levels of 2555 mouse miRNA probes using liver tissue and serum samples from the control and ipragliflozin groups. As shown in Table 1, three miRNAs were significantly upregulated and three miRNAs were downregulated in liver tissue samples of the ipragliflozin group compared with those of the control group. Unsupervised hierarchical clustering analysis using Pearson’s correlation coefficient showed that the miRNA profile of the ipragliflozin group clustered separately from that of the control group in the liver tissue (Fig 4A). In addition, 59 miRNAs were significantly upregulated and 84 miRNAs were downregulated in serum samples of the ipragliflozin group (Table 2). Unsupervised hierarchical clustering analysis using Pearson’s correlation coefficient showed that the serum miRNA profile of the ipragliflozin group clustered separately from that of the control group (Fig 4B). Among these differentially expressed miRNAs after ipragliflozin treatment, only miR-19b-3p was commonly altered in both the liver tissue and serum samples (Fig 4C). In addition, RT-qPCR analysis confirmed that miR-19b-3p expression in the serum was significantly upregulated in the ipragliflozin group (Fig 4D).

**Table 1:** miRNA expression profile in the liver tissue of mice treated with ipragliflozin.

NAME	Expression	P values	FC
mmu-let-7f-5p	up	0.015	1.31
mmu-miR-19b-3p	down	0.046	0.73
mmu-miR-194-2-3p	down	0.036	0.41
mmu-miR-338-3p	up	0.042	1.89
mmu-miR-1224-5p	down	0.003	0.6
mmu-miR-6239	up	0.003	1.55

**Table 2:** miRNA expression profile in the serum of mice treated with ipragliflozin.

NAME	Expression	P values	FC
mmu-let-7d-5p	up	0.032465347	2.483881463
mmu-let-7i-5p	up	0.035107402	2.688676672
mmu-miR-10b-5p	down	0.017432574	0.512489943
mmu-miR-15a-5p	up	0.015998853	4.110070512
mmu-miR-15b-5p	up	0.007784478	3.114517691
mmu-miR-17-5p	up	0.011446966	3.275500008
mmu-miR-19b-3p	up	0.012824614	2.457303814
mmu-miR-20a-5p	up	0.007513239	3.36114997
mmu-miR-20b-5p	up	0.009565522	2.88105741
mmu-miR-21a-5p	up	0.033177456	2.10672135
mmu-miR-29b-1-5p	down	0.020390635	0.21859113
mmu-miR-29b-2-5p	down	0.012661728	0.451819206
mmu-miR-92a-2-5p	up	0.026840788	1.537022077
mmu-miR-103-3p	up	0.040349713	2.16310064
mmu-miR-106a-5p	up	0.015896854	3.01872662
mmu-miR-106b-5p	up	0.006146515	3.600172973
mmu-miR-107-3p	up	0.014776766	2.245604513
mmu-miR-125b-1-3p	up	0.00525236	1.765736441
mmu-miR-130b-5p	up	0.019956367	1.886789399
mmu-miR-153-5p	down	0.036001941	0.524635254
mmu-miR-181c-3p	down	0.013905708	0.644067175
mmu-miR-182-5p	down	0.00578376	0.567662095
mmu-miR-185-5p	up	0.01894911	2.025154565
mmu-miR-195a-5p	up	0.037139597	1.768431788
mmu-miR-199b-5p	up	0.003642907	1.771450276
mmu-miR-203-3p	up	0.040860233	1.963198145
mmu-miR-211-3p	down	0.002273896	0.600382974
mmu-miR-216c-3p	up	0.021245251	1.504895698
mmu-miR-292b-5p	down	0.040322164	0.530992939
mmu-miR-298-3p	down	0.042619849	0.624957444
mmu-miR-301b-5p	up	0.015954105	1.579136801
mmu-miR-320-5p	down	0.005878316	0.654749079
mmu-miR-322-5p	up	0.035902124	1.727071795
mmu-miR-325-3p	up	0.024831012	1.578269836
mmu-miR-327	down	0.022937449	0.527533926
mmu-miR-330-5p	down	0.043312635	0.606184046
mmu-miR-350-3p	up	0.014334686	1.70125942
mmu-miR-361-3p	up	0.026653481	1.646891919
mmu-miR-370-3p	up	0.010943872	1.633447966
mmu-miR-375-5p	down	0.002804536	0.569350632
mmu-miR-378a-3p	up	0.045121104	1.657932082
mmu-miR-433-3p	down	0.029222719	0.453496704
mmu-miR-451a	up	0.008487654	3.208635799
mmu-miR-494-3p	up	0.000518269	3.93445192
mmu-miR-503-5p	up	0.040122118	2.080671214
mmu-miR-541-3p	down	0.024678192	0.553633771
mmu-miR-542-5p	up	0.00253207	1.78329934
mmu-miR-546	down	0.007829572	0.653734419
mmu-miR-551b-3p	down	0.029156194	0.583479548
mmu-miR-598-5p	down	0.008913331	0.616797243
mmu-miR-705	up	0.002033938	1.832202093
mmu-miR-711	up	0.002922611	1.967604808
mmu-miR-770-3p	down	0.03743399	0.649882435
mmu-miR-871-3p	down	0.002738532	0.605548384
mmu-miR-1190	up	0.041934895	1.923090721
mmu-miR-1195	down	0.018618701	0.312928155
mmu-miR-1224-3p	up	0.026759263	1.542855166
mmu-miR-1258-3p	down	0.010013318	0.539595652
mmu-miR-1298-3p	down	0.011535536	0.421378304
mmu-miR-1306-5p	down	0.000136003	0.437651558
mmu-miR-1892	up	0.002575517	2.400178876
mmu-miR-1897-5p	down	0.000310438	0.398489125
mmu-miR-1927	down	0.001785406	0.440648913
mmu-miR-1932	down	0.024479857	0.474963679
mmu-miR-1964-3p	down	0.009764985	0.483050878
mmu-miR-3063-5p	down	0.002074134	0.627758418
mmu-miR-3080-5p	down	0.024081211	0.562615297
mmu-miR-3083-5p	down	0.002625044	0.582607074
mmu-miR-3093-5p	down	0.045559682	0.414761485
mmu-miR-3094-5p	down	0.03551582	0.550637818
mmu-miR-3097-5p	down	0.030136625	0.595396348
mmu-miR-3099-5p	down	0.014994128	0.605578856
mmu-miR-3102-5p	up	0.000272639	3.583244049
mmu-miR-3572-3p	up	0.012279693	1.666708206
mmu-miR-3618-5p	down	0.002399786	0.589498909
mmu-miR-3970	down	0.004959244	0.297417499
mmu-miR-5108	down	0.010646173	0.417474915
mmu-miR-5112	down	0.013960104	0.452278713
mmu-miR-5119	down	0.007637071	0.23833
mmu-miR-5124a	down	0.031679083	0.60722669
mmu-miR-5130	down	0.01137853	0.449324032
mmu-miR-5624-5p	up	0.024709001	1.792079721
mmu-miR-5627-3p	down	0.010188442	0.623937412
mmu-miR-6351	up	0.021949697	2.070420414
mmu-miR-6357	up	0.037570764	1.588171452
mmu-miR-6367	up	0.017647359	2.147853923
mmu-miR-6378	up	0.027409417	2.899717799
mmu-miR-6379	down	0.037943544	0.423130637
mmu-miR-6385	down	0.008893543	0.335629517
mmu-miR-6388	up	0.004000261	3.104101661
mmu-miR-6391	up	0.026501885	2.376920434
mmu-miR-6403	down	0.001200351	0.303564453
mmu-miR-6405	down	0.018997432	0.573852937
mmu-miR-6416-3p	down	0.008051056	0.353181923
mmu-miR-6538	down	0.031427327	0.536437134
mmu-miR-6769b-5p	up	0.001971381	2.40554815
mmu-miR-6910-5p	down	0.003988466	0.457922782
mmu-miR-6912-5p	down	0.00690221	0.427355946
mmu-miR-6913-5p	down	0.002762037	0.434121664
mmu-miR-6914-5p	up	0.043518418	2.241916586
mmu-miR-6915-5p	down	0.026228015	0.664564476
mmu-miR-6917-5p	down	0.024773315	0.265390164
mmu-miR-6919-5p	down	0.000395826	0.339718827
mmu-miR-6932-5p	down	0.030370253	0.35404213
mmu-miR-6934-5p	up	0.006006173	1.631106041
mmu-miR-6935-3p	down	0.012046036	0.588538438
mmu-miR-6936-5p	down	0.019503392	0.391604276
mmu-miR-6937-5p	up	0.007879246	1.794443094
mmu-miR-6941-5p	down	0.007775757	0.489934788
mmu-miR-6979-5p	down	0.007595022	0.624796231
mmu-miR-6984-5p	down	0.012035957	0.58578775
mmu-miR-6986-5p	down	0.009529806	0.447395518
mmu-miR-6990-5p	down	0.000378271	0.569237342
mmu-miR-6991-5p	up	0.04317317	1.824456214
mmu-miR-6994-5p	down	0.026004142	0.476611184
mmu-miR-6995-5p	up	0.039619129	2.774726807
mmu-miR-6997-5p	up	0.026754222	2.569162647
mmu-miR-7001-5p	down	0.005262089	0.611341653
mmu-miR-7009-5p	down	0.012553863	0.519167855
mmu-miR-7014-5p	down	0.001055284	0.522538427
mmu-miR-7023-5p	down	0.015298511	0.550255277
mmu-miR-7030-5p	up	0.01974348	1.941177594
mmu-miR-7031-5p	up	0.039365782	2.162341052
mmu-miR-7042-5p	up	0.011729098	5.03857355
mmu-miR-7046-3p	down	0.008557471	0.571278198
mmu-miR-7072-5p	down	0.009715702	0.662675028
mmu-miR-7078-5p	down	0.027151776	0.577215293
mmu-miR-7115-5p	up	0.046881619	2.374831118
mmu-miR-7232-3p	up	0.008515991	1.589876191
mmu-miR-7238-5p	down	0.034519749	0.588725971
mmu-miR-7242-5p	down	0.014665521	0.664703556
mmu-miR-7647-3p	up	0.042826133	1.968581299
mmu-miR-7648-3p	down	0.039672759	0.48976441
mmu-miR-7653-3p	down	0.000707897	0.126814976
mmu-miR-7666-3p	down	0.015431022	0.405453459
mmu-miR-7669-3p	down	0.01482687	0.309926482
mmu-miR-7672-5p	down	0.004390638	0.346143601
mmu-miR-7686-3p	down	0.001391195	0.42803959
mmu-miR-7687-5p	down	4.80E-05	0.61551571
mmu-miR-8093	down	0.000517494	0.662544381
mmu-miR-8109	down	0.048621637	0.588998681
mmu-miR-8112	down	0.024519221	0.573434916
mmu-miR-8114	down	0.015382228	0.589988567

## 3. Discussion

The prevalence of NAFLD and NASH has increased worldwide [35–37]. T2DM is an important risk factor for NAFLD; Matteoni et al. [38] demonstrated that 33–50% of T2DM patients have NAFLD. Although the antidiabetic drug pioglitazone, which is an insulin-sensitizing agent, has a beneficial effect for NASH, long-term outcomes about its efficacy and safety, including the side effects of cardiovascular disease, congestive heart failure, bladder cancer, and bone loss, have not yet been elucidated [39]. To date, no effective drug for NASH has been discovered; thus, it is critical to investigate an effective therapy for patients with NASH. Therefore, we explored the effects of ipragliflozin on NASH and the underlying mechanism using STAM mice.

Our results demonstrated that ipragliflozin had a therapeutic effect on NASH in STAM mice. Ipragliflozin has been reported to improve hyperglycemia by inducing the urinary excretion of glucose [40, 41]. Ipragliflozin has been shown to dose-dependently increase urinary glucose excretion in various types of mice, including normal and streptozotocin–nicotinamide-induced diabetic mice [25–27, 42]. In the present study, ipragliflozin improved the lobular inflammation score, although our STAM mouse model is an insulin-deficient NASH model. Hence, we hypothesize that ipragliflozin improved hyperglycemia, liver inflammation, and liver fibrosis through not only urinary glucose excretion but also other mechanisms. Reactive oxygen species (ROS) are recognized as an origin of oxidative stress and produced through the metabolism of free fatty acids in mitochondria, microsomes, and peroxisomes. Migration of the electrons supplied to the mitochondrial respiratory chain eventually reach the cytochrome C oxidase, combining with oxygen and protons to form water [43]. However, some of these electrons leak out and form the superoxide anion radical. SOD2 dismutes this radical into hydrogen peroxide, which is detoxified into water by GPXS and CAT [44]. Low levels of ROS are associated with important cellular processes. Therefore, proper control of oxidative stress and a balance between oxidative and antioxidative responses are pivotal [45]. Excess superoxide can be produced in damaged mitochondria by electron leakage and this excess is converted to hydrogen peroxide by SOD2. Although GPXS or CAT can metabolize hydrogen peroxide to nontoxic H_2_O, iron-mediated Fenton and/or Haber-Weiss reactions produce highly reactive and toxic hydroxyl radicals. The levels of iron which is an inducer of oxidative stress are increased in NASH. Reduced iron levels brings about the improvement of liver functions in patients with chronic liver diseases [46]. In our present study, SOD2 and CAT expression was upregulated in the liver after ipragliflozin administration (Fig 3A and 3B). Thus, most mitochondrial ROS might be detoxified by ipragliflozin. These data suggest that ipragliflozin might inhibit NASH development in the liver, reinforcing the activity of the key ROS-scavenging antioxidant enzymes SOD2 and CAT directly or indirectly.

Furthermore, ipragliflozin is said to inhibit SGLT1 and SGLT2. Since the intestinal and hepatic axes are known to play an important role in NAFLD/NASH, we also examined the expression of SGLT1 and SGLT2 in the intestinal cells of STAM mice. As a result, the expression of SGLT1 was upregulated, but the expression of SGLT2 was not expressed in small intestinal cells (S4 Fig). Surprisingly, ipragliflozin treatment resulted in the expression of p21 in intestinal cells in vitro and in vivo (S5 and S6 Figs). These results suggest that ipragliflozin may be involved in the proliferation of intestinal cells, resulting in changes in enterohepatic immunity. Surprisingly, SGLT2 was not expressed in normal liver, but was expressed in various HCC cell lines (S7 Fig). Therefore, our results suggest that ipragliflozin does not act directly on the normal liver, but inhibits the development of NASH by suppressing SGLT2 in the kidney and SGLT1 in the small intestine.

Among the upregulated miRNAs identified in the serum, miR-19b-3p was found to be significantly downregulated in the liver following ipragliflozin treatment in STAM mice; however, miR-34a-5p, miR-149-5p, and miR-122-5p were not changed in the serum (S2 Fig) and liver tissue (S3 Fig). There are several potential reasons for the increase of miR-19b-39 in the serum and its decrease in the liver, including (i) leaked miR-19b-3p increased due to the destruction of hepatocytes and (ii) miR-19b-3p was secreted from hepatocytes as a circulating miRNA. Recently, serum miR-19b-3p up-regulation was shown to alleviate the lipopolysaccharide (LPS)-induced inflammatory response of human umbilical vein endothelial cells, thereby inhibiting the LPS-induced expression of the pro-inflammatory cytokines interleukin (IL)-6 and tumor necrosis factor (TNF)-α [47]. In addition, Duan et al. [48] demonstrated that miR-19b-3p attenuated IL-1β-induced extracellular matrix degradation and inflammatory injury. These reports suggest that ipragliflozin might have attenuated NASH development through not only the improvement of hyperglycemia by inducing the urinary excretion of glucose but also by modulation of miR-19b-3p to inhibit IL-6 and TNF-α expression. Furthermore, circulating miR-19b-3p has been reported to be involved in liver fibrosis via inhibition of C–C motif chemokine receptor (CCR2), indicating that up-regulation of circulating miR-19b-3p suppresses liver fibrogenesis via the regulation of inflammation [49].

In conclusion, we confirmed that ipragliflozin improves NASH development and identified a new mitochondrial pathway and a key miRNA that were modulated by ipragliflozin in a NASH mouse model. Our findings might provide a simple and novel therapeutic strategy for NASH.

## 4. Materials and methods

### 4.1. Animals and experimental design

STAM mice, a NASH-cirrhosis-hepatocarcinogenic model, were purchased from Stelic Institute & Co., Inc. (Tokyo, Japan). This mouse model progresses from NAFLD to NASH at 8 weeks of age and develops HCC at 16 weeks of age [50]. In brief, for model establishment, C57BL/6J male mice were injected with 20 μL solution (10 mg/mL) of streptozotocin (Sigma-Aldrich Japan, Tokyo, Japan) 2 days after birth to destroy pancreatic beta cells, and were subsequently fed a high-fat diet (HFD-32; CLEA-Japan, Tokyo, Japan). For NASH experiments, 5-week-old male STAM mice were divided into two experimental groups: the control group was fed the HFD and the ipragliflozin group was fed the HFD mixed with 16.7 μg/day of ipragliflozin (Astellas Pharma Inc., Tokyo, Japan) for 5 weeks. The feed was replaced 3 times per week, so that it was kept dry, as HFDs tend to retain moisture. After 10 weeks, the mice were sacrificed using carbon dioxide. Blood samples were obtained from the right atrium by cardiac puncture and the livers were excised. The livers were cut into pieces and fixed in 10% formalin for histological analysis or fresh-frozen in liquid nitrogen and stored at –80°C in a freezer until use. The animals had free access to water and food and were maintained under specific pathogen-free conditions in a temperature-controlled animal facility with a 12-h light-dark cycle. All protocols and procedures conformed to the guidelines of the Kagawa University Committee for Care and Use of Laboratory Animals and were approved by the Animal Experiments Ethics Committee of Kagawa University (approved number 15145). This study was carried out in compliance with the ARRIVE guidelines.

### *4*.2. Measurement of fasting blood glucose, plasma aminotransferases, and triglycerides

The levels of fasting blood glucose were measured after 12 h of fasting using a blood glucose meter (GT1820; Arkray Inc., Kyoto, Japan). Aspartate aminotransferase (AST), alanine aminotransferase (ALT), and alkaline phosphatase (ALP) levels were determined by standard methods at Shikoku Chuken Central Biolaboratory (Takamatsu, Japan).

### 4.3. Histological analysis of the liver

Sections of formalin-fixed livers were used for hematoxylin-eosin and Masson’s trichrome staining for pathological analysis. NAFLD activity was assessed by the NAFLD activity score, as described by Kleiner et al., with separate scores for steatosis (0–3), hepatocellular ballooning (0–2), and lobular inflammation (0–3) [51]. All liver specimens were assessed by two hepatologists (AM and TM) blinded to the identities of the study groups.

### 4.4. Reverse transcription-quantitative polymerase chain reaction (RT-qPCR)

The whole RNA extracted from murine liver using RNeasy kit (Qiagen) was used for RT-qPCR with manufacturer’s directions. Each extracted RNA was reverse-transcribed with a TaqMan Reverse Transcription kit (Life Technologies Japan) and quantified using TaqMan Gene Expression Assay (Applied Biosystems, Foster City, USA) with the manufacturer’s directions. We analyzed the relative quantification of targeting mRNAs by using the comparative cycle threshold (CT) method (2^−ΔΔCT^). *Actb* was quantified as a house keeping gene.

Circulating miRNA purified from murine serum using the QIAGEN miRNeasy serum-plasma kit (Qiagen K.K., Tokyo) and tissue miRNA extracted from murine liver using the miRNeasy Mini Kit (Qiagen, Hilden, Germany) were used for RT-qPCR with the manufacturer’s directions. Each extracted miRNA was reverse-transcribed with TaqMan MicroRNA Reverse Transcription kit (Life Technologies Japan, Tokyo). For the relative quantification of serum miRNA, *Caenorhabditis elegans* miR-39 (cel-miR-39) was spiked in each sample as an external control. The serum miRNA was quantified using the TaqMan MicroRNA assay with the manufacturer’s directions. We analyzed the relative quantification of targeting miRNAs by using the comparative cycle threshold (CT) method (2^−ΔΔCT^) with spiked cel-miR-39 serving as an external control. For the quantification of liver tissue miRNA, *Rnu6b* was used as an internal control for relative quantification.

### 4.5. miRNA array

Whole miRNA extracted from the serum or liver tissues using a miRNeasy Mini Kit (Qiagen, Hilden, Germany) with the manufacturer’s directions was determined by an Agilent 2100 Bioanalyzer (Agilent Technologies, Santa Clara, CA, USA). The miRNA samples were measured using RNA 6000 Nano Kit (Agilent Technologies) and labeled by miRCURYHy3/Hy5 Power Labeling Kit. Subsequently these miRNAs were hybridized to a mouse miRNA Oligo chip (v. 21.0; Toray Industries, Tokyo, Japan). Hybridized miRNAs were detected using 3D-Gene Scanner 3000 (Toray Industries) and analyzed by 3D-Gene extraction version 1.2 software (Toray Industries). Comprehensive microarray analysis of miRNA expression between the control and ipragliflozin groups were performed using GeneSpring GX 10.0 software (Agilent Technologies). We used quantile normalization for making the background identical. Differentially expressed miRNAs were verified using the Mann-Whitney U test and the furthest-neighbor method with the absolute uncentered Pearson’s correlation coefficient as a metric was used for hierarchical clustering. The base-2 logarithm of the intensity was median-centered for each row in a heat map which was created using the relative expression intensity for each miRNA.

### 4.6. Statistical analysis

All analyses were conducted using computer-assisted GraphPad Prism 8.4.2 (GraphPad Software, San Diego, CA, USA). Paired analysis between the groups was conducted using Student’s *t* test. *P* < 0.05 was considered to indicate a significant difference between the groups.

## 5. Conclusions

Our present study demonstrated that ipragliflozin improved NASH development and identified a new mitochondrial pathway and a key miRNA that were regulated by ipragliflozin in a NASH mouse model. Ipragliflozin also affected to the small intestine for preventing NASH development. Therefore, our findings might provide a novel therapeutic strategy for NASH.

## Supporting information

S1 Fig Ipragliflozin attenuated liver fibrosis using NAFLD fibrosis score. (TIF)

S2 Fig SGLT1 and SGLT2 expression in the small intestine. (TIF)

S3 Fig p21 mRNA expression by ipragliflozin in small intestine cancer cell line. (TIF)

S4 Fig p21 expression in the small intestine between Control and Ipragliflozin group. (TIF)

S5 Fig Microarray data analysis revealed that miR-34a-5p, miR-149-5p, and miR-122-5p expressions were not significantly changed in the serum of STAM mice treated with ipragliflozin. (TIF)

S6 Fig Microarray data analysis revealed that miR-34a-5p, miR-149-5p, and miR-122-5p expressions were not significantly changed in the liver of STAM mice treated with ipragliflozin. (TIF)

S7 Fig SGLT2 expression in hepatocellular carcinoma cell lines. (TIF)

S1 File (XLSX)

S2 File (XLSX)

S3 File (XLSX)

S4 File (XLSX)

S5 File (XLSX)

S6 File (XLSX)

S7 File (XLSX)

S8 File (XLSX)

S9 File (XLSX)
