# Supplementary figures and images for: Ipragliflozin attenuates non-alcoholic steatohepatitis development in an animal model
Source: PLoS One. 2022 Feb 22;17(2):e0261310. doi: 10.1371/journal.pone.0261310 (PMC8863244; doi:10.1371/journal.pone.0261310)

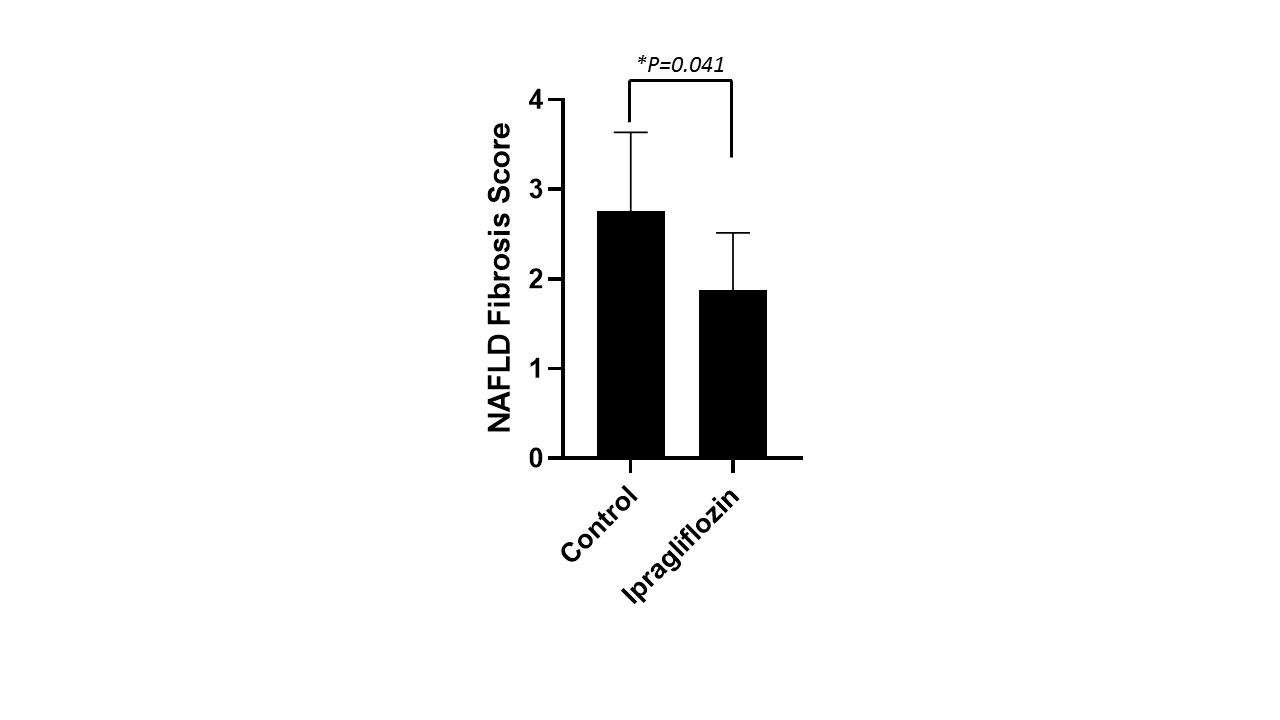

Supplement: S1 Fig — (TIF) [file pone.0261310.s001.tif]

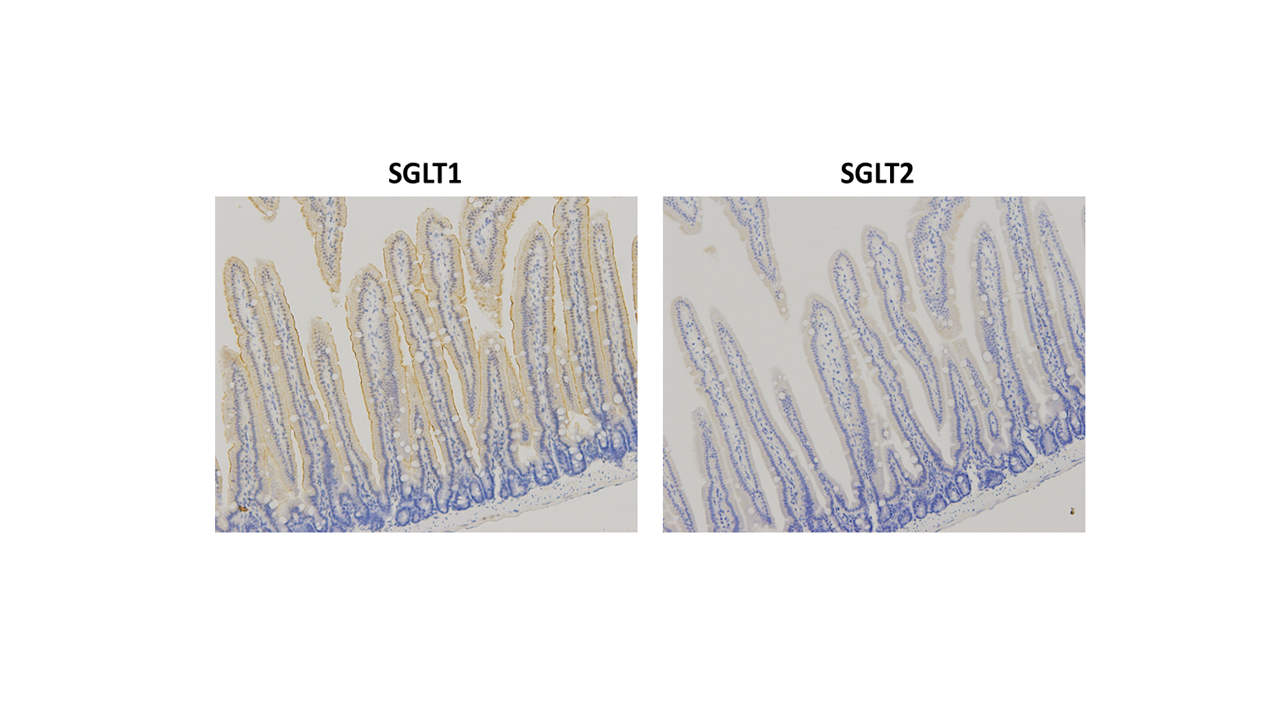

Supplement: S2 Fig — (TIF) [file pone.0261310.s002.tif]

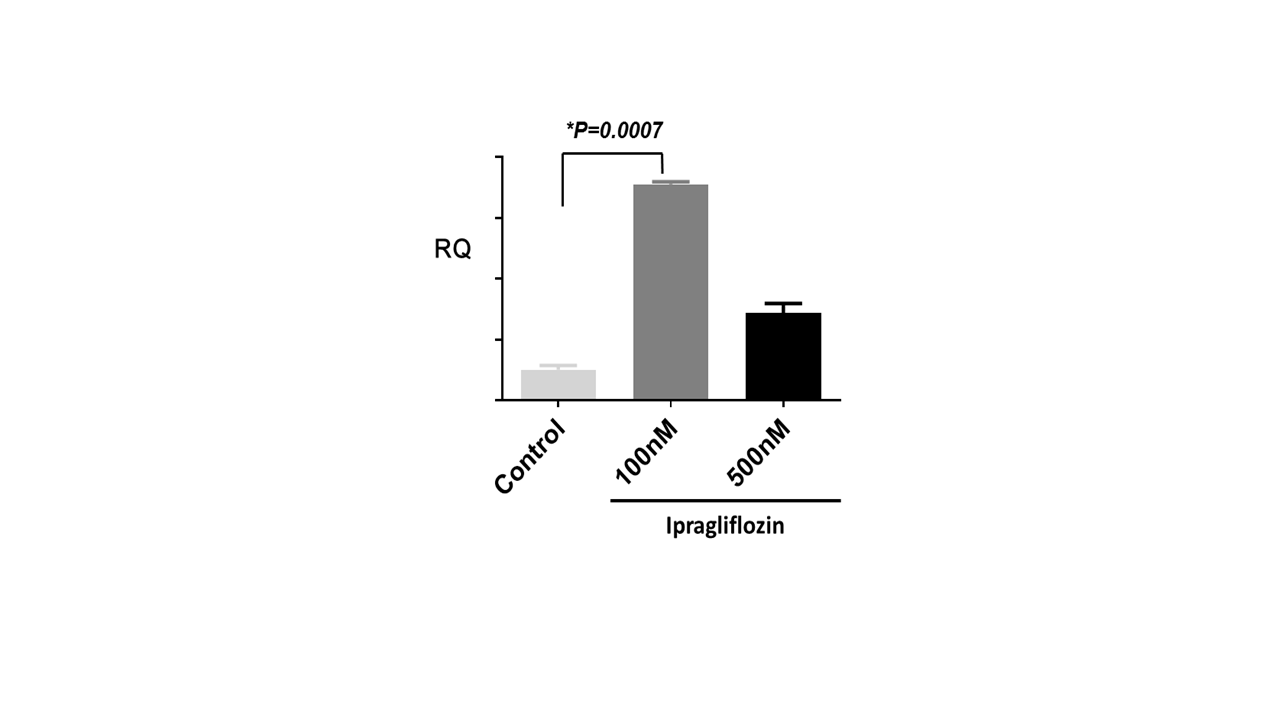

Supplement: S3 Fig — (TIF) [file pone.0261310.s003.tif]

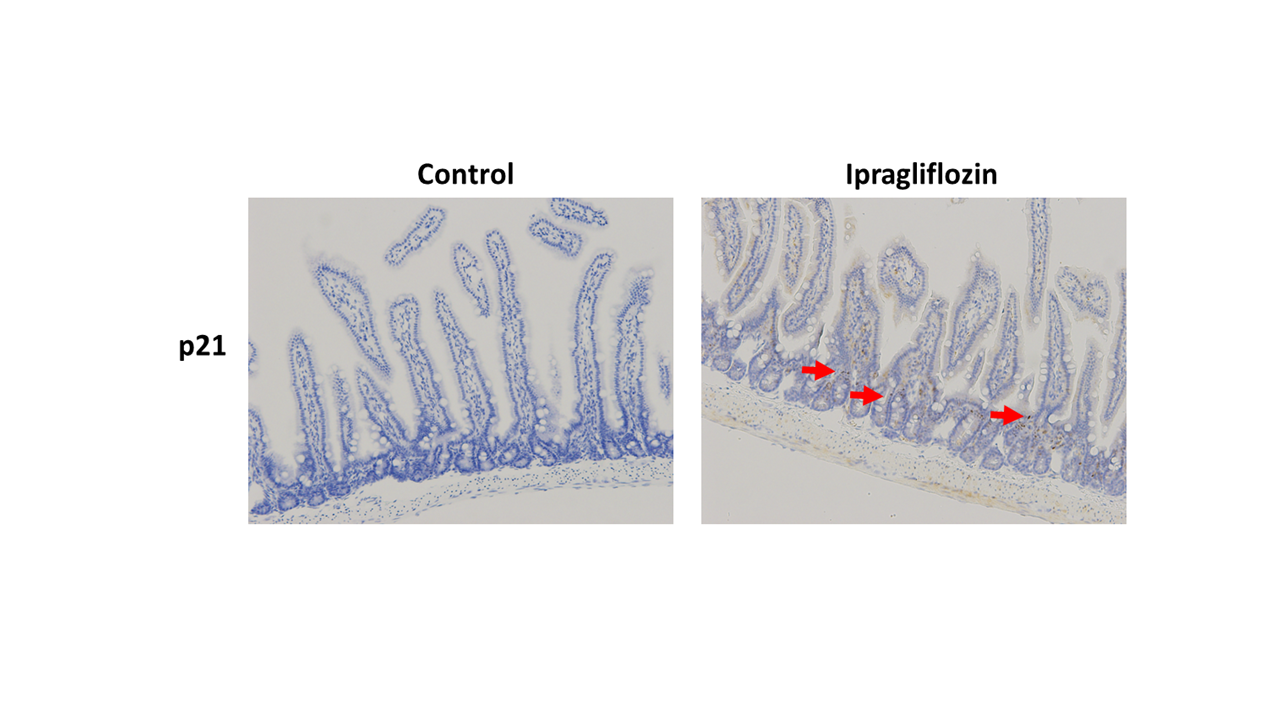

Supplement: S4 Fig — (TIF) [file pone.0261310.s004.tif]

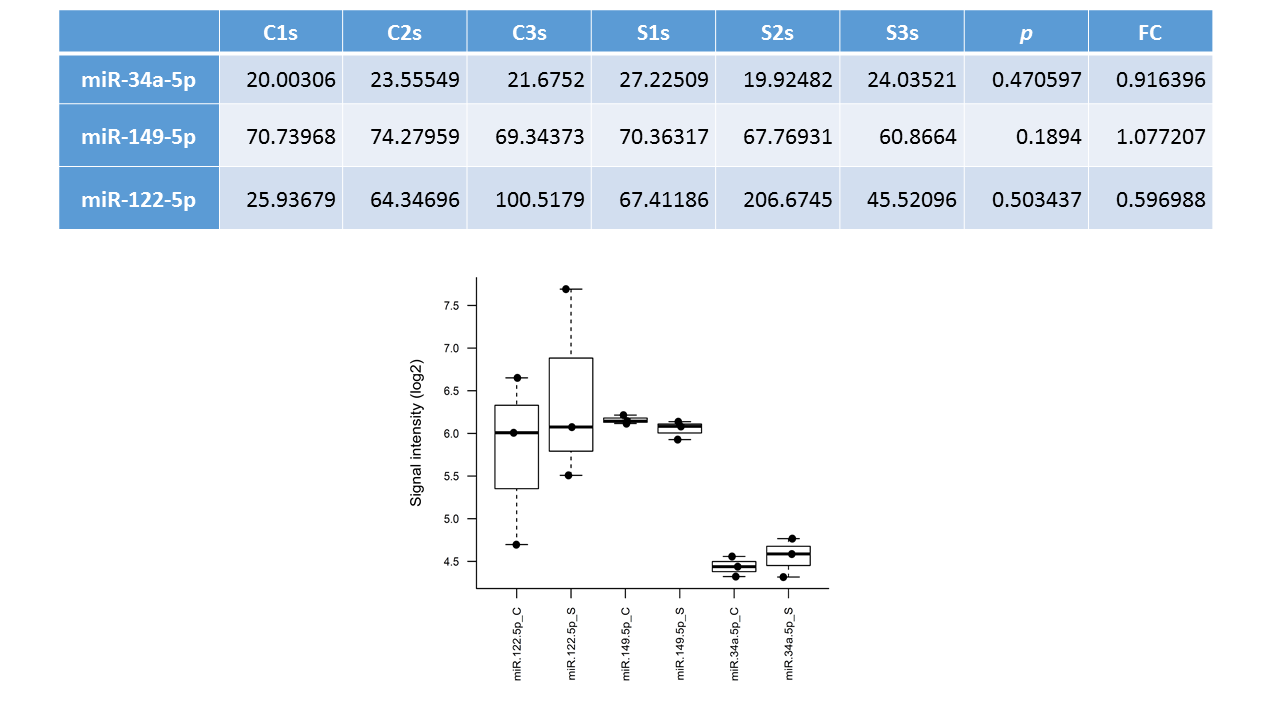

Supplement: S5 Fig — (TIF) [file pone.0261310.s005.tif]

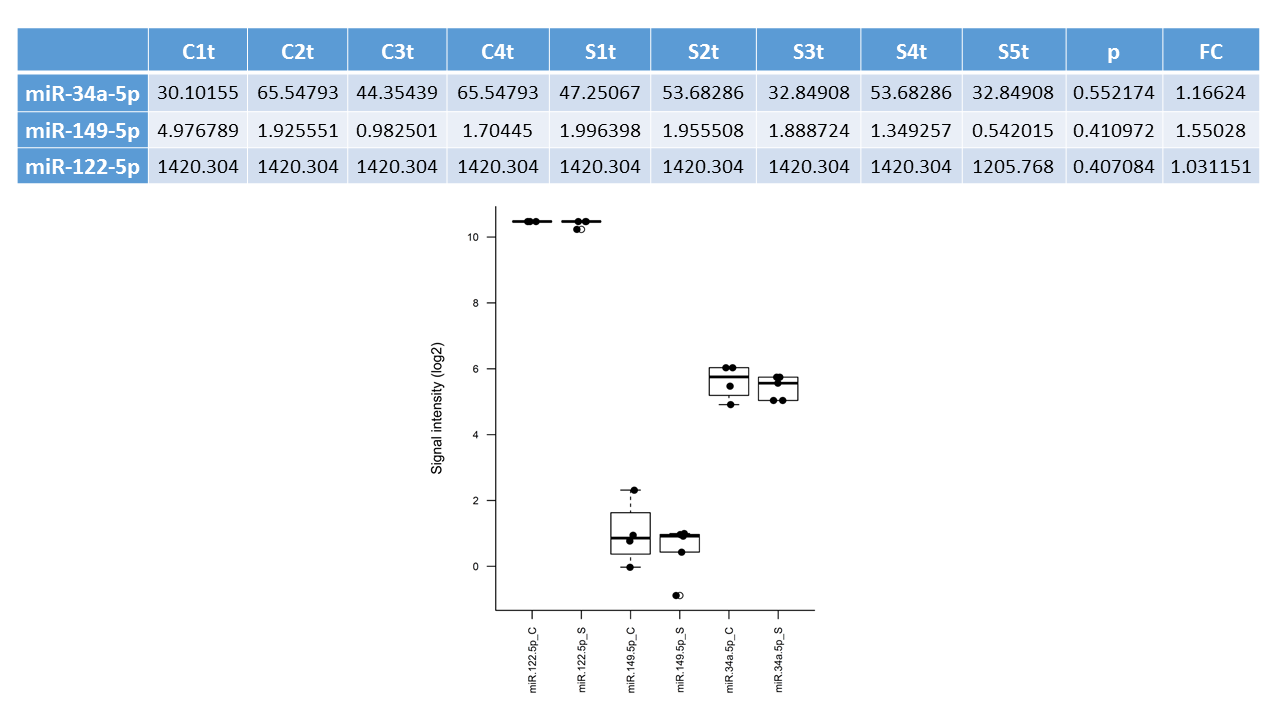

Supplement: S6 Fig — (TIF) [file pone.0261310.s006.tif]

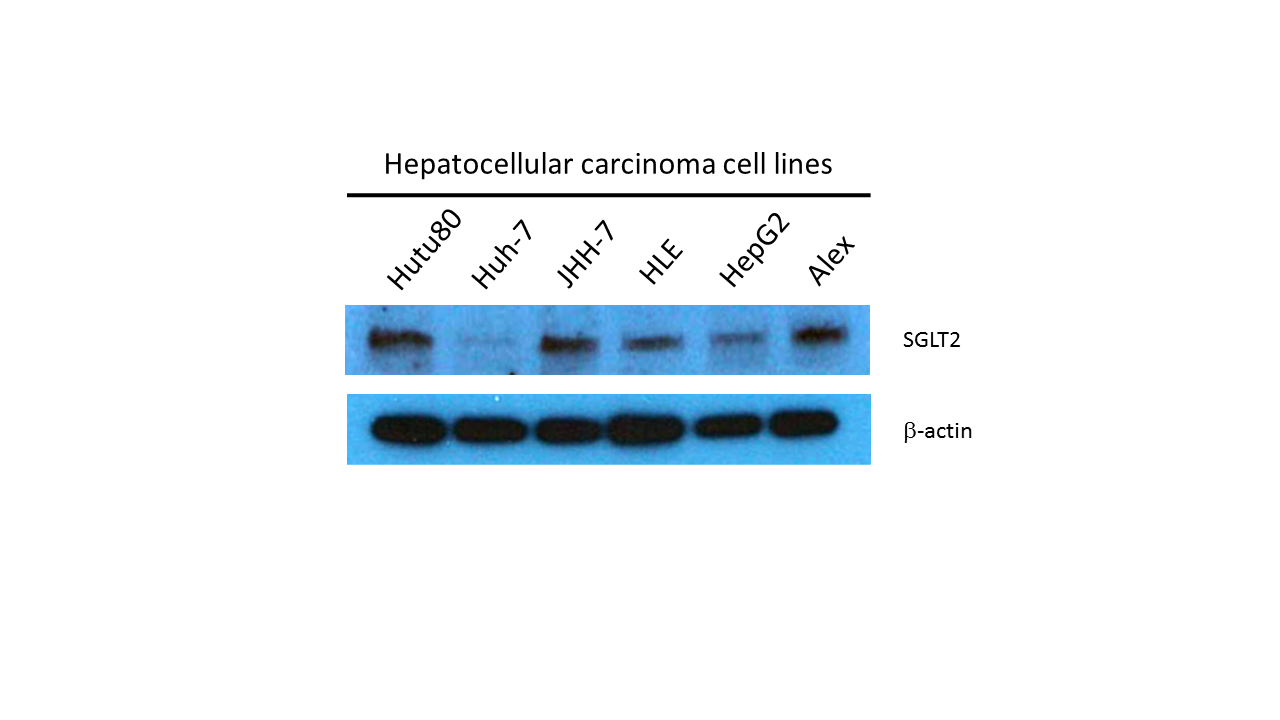

Supplement: S7 Fig — (TIF) [file pone.0261310.s007.tif]
